# Development of Renal Denervation Catheter Based on Piezoelectric Ultrasonic Transducer

**DOI:** 10.3390/mi17101207

**Published:** 2026-10-21

**Authors:** Qian Li, Tao Zhou, Dan Qin, Yongliang Wu, Xiang Li, Song Li, Xiong Chen, Zhipeng Wu

**Affiliations:** 1 Acoustic Life Science Co., Ltd., Shanghai 201203, China; qian.li@als-ultrasound.com (Q.L.); tao.zhou@als-ultrasound.com (T.Z.); dan.qin@als-ultrasound.com (D.Q.); yongliang.wu@als-ultrasound.com (Y.W.); xiang.li@als-ultrasound.com (X.L.); 2 College of Biomedical Engineering and Instrument Science, Zhejiang University, Hangzhou 310027, China; 3 Sonoscape Medical (Wuhan) Co., Ltd., Wuhan 430074, China; lis1@sonoscape.net; 4 Sonoscape Medical Corp., Shenzhen 518107, China

**Keywords:** piezoelectric ultrasonic transducer, renal denervation, balloon catheter

## Abstract

In this manuscript, a miniaturized ultrasonic Renal Denervation (RDN) catheter for removing sympathetic nerves around the renal artery is proposed. The RDN catheter consists of an ultrasonic transducer, a balloon catheter and a control system. Owing to the use of a piezoelectric ceramic tube as the ultrasonic transducer, the RDN catheter is designed to provide 360° circumferential acoustic exposure around the renal artery. The piezoelectric ceramic tube has an active length of 6 mm, an inner diameter of 1.1 mm, and an outer diameter of 1.6 mm and the material of piezoelectric ceramic tube is PZT8. The balloon catheter of the proposed RDN catheter is designed and optimized so that the diameter of the portion inserted into the vessel is ≤1.8 mm, with the aim of enabling access to the renal artery via Transradial Access (TRA). In addition, a liquid circulation system is integrated into the balloon catheter to cool the ultrasonic transducer, with the aim of limiting heating near the renal artery wall. The measured resonant and anti-resonant frequencies of the ultrasonic transducer are 8.72 MHz and 9.69 MHz, respectively. Under 70 W input electrical excitation at 9.69 MHz, the average acoustic pressure measured at 6–7 mm from the catheter surface is 2.11 MPa. In addition, the control system prototype is also developed. Finally, the thermal feasibility of the proposed catheter is evaluated through phantoms and ex vivo tissues. In the phantom, visible whitening appears by 0.5 s, and an annular whitened region is evident by 8 s; at 15 s, its image-estimated thickness is approximately 3.6 mm, with an adjacent transparent interval of approximately 1.2 mm. Ex vivo porcine liver also shows macroscopic discoloration consistent with thermal denaturation. These observations support the preliminary acoustic and thermal feasibility of the proposed RDN catheter.

## 1. Introduction

According to the statistical data of the World Health Organization, an estimated 1.4 billion adults aged 30–79 years worldwide had hypertension in 2024, which represents 33% of the population in this age range. Hypertension is a major cause of premature death worldwide [1]. Most of the blood pressure of the hypertensive patients can be controlled through antihypertensive medications. Unfortunately, Resistant Hypertension occurs in 5–30% of hypertensive patients [2,3]. Resistant Hypertension is defined as a chronic disease characterized by using three or more antihypertensive medications (one of which is a diuretic) that fail to lower the blood pressure to the target levels of 140/90 mmHg [2,4]. The excessive activity of the renal sympathetic nerves is one of the contributing factors to Resistant Hypertension. Accordingly, removal of some renal sympathetic nerves is a feasible therapeutic method for treating Resistant Hypertension [2,5,6,7,8,9,10]. The Renal Denervation (RDN) catheter is the main approach to achieving the removal of renal sympathetic nerves via physical or chemical methods, which uses a percutaneous access route to the renal artery.

Currently, three main types of RDN catheters are used in clinical practice: the radiofrequency RDN catheter, the ultrasonic RDN catheter and the neurotoxin-injected RDN catheter [11]. The radiofrequency RDN catheter uses a catheter to generate a medium-frequency alternating current via several electrodes. The electrodes are in contact with the renal artery wall. Then, the heat energy produced by the medium-frequency alternating current in the tissue around the renal artery can pass through the renal artery wall to the nerves surrounding the renal artery. The neurotoxin-injected RDN catheter delivers neurotoxins (typically a total of 0.6 mL of absolute ethanol) via microneedles to the nerves surrounding the renal artery. The ultrasonic RDN catheter emits ultrasonic energy to heat the renal sympathetic nerves, leading to the denaturation of sympathetic nerves. Since the transmission of ultrasonic waves does not require the ultrasonic transducer of the ultrasonic RDN catheter to be in direct contact with the renal artery wall, the ultrasonic RDN catheter can be designed as a balloon catheter, and the ultrasonic transducer can be mounted inside the balloon catheter. The balloon catheter isolates the ultrasonic transducer from the renal arterial wall, with the aim of reducing thermal damage to the renal artery wall induced by the transducer, which serves as the main heat source. In addition, the circulating liquid inside the balloon catheter provides cooling for the transducer. Compared with the radiofrequency RDN catheter and the neurotoxin-injected RDN catheter, the ultrasonic RDN catheter is a relatively safe RDN catheter because it does not easily cause thermal damage to the renal artery wall and is non-toxic.

At present, many efforts are focused on the radiofrequency RDN catheter, which include the design and therapeutic effect [12,13,14,15,16,17]. Furthermore, many radiofrequency RDN catheter products are available on the market, such as Symplicity Spyral [18], Netrod [19,20], etc. However, there are few research papers on the ultrasonic RDN catheter, and most of them focus on the therapeutic efficacy of denervation [9,21,22,23,24,25,26]. There are also relatively few commercial ultrasonic RDN catheters, among which the ReCor Medical Paradise system is one of the well-known commercial products [27]. These reports establish relevant prior art and therapeutic context. Device-level engineering reports that connect transducer dimensions, electrical loading, catheter assembly, and acoustic characterization remain useful for developing and evaluating compact research prototypes.

This manuscript proposes an ultrasonic RDN catheter for removing renal artery sympathetic nerves, which consists of an ultrasonic transducer, a balloon catheter, and a control system. The piezoelectric ceramic tube is selected as the ultrasonic transducer for the proposed ultrasonic RDN catheter. According to the requirements of denervation therapy and access to the renal artery through Transradial Access (TRA), the material and dimensions of the piezoelectric ceramic tube are designed. The performance of the selected piezoelectric ceramic tube is characterized, showing a resonant frequency of 8.72 MHz and an anti-resonant frequency of approximately 9.69 MHz. The output acoustic field is also characterized, achieving an acoustic pressure of 2.11 MPa at a distance of 6–7 mm from the tube at an anti-resonant frequency. The balloon catheter of the proposed ultrasonic RDN is designed, and the RDN control system prototype is also developed. Finally, the thermal feasibility of the proposed catheter is evaluated through phantoms and ex vivo tissues. The comparison between the proposed RDN catheter and the prior art is summarized in Table 1. Although the proposed RDN catheter is still in the prototype stage, its miniaturized design is intended to support TRA delivery to the renal artery.

## 2. Design of the Ultrasonic RDN Catheter

**Figure 1:**
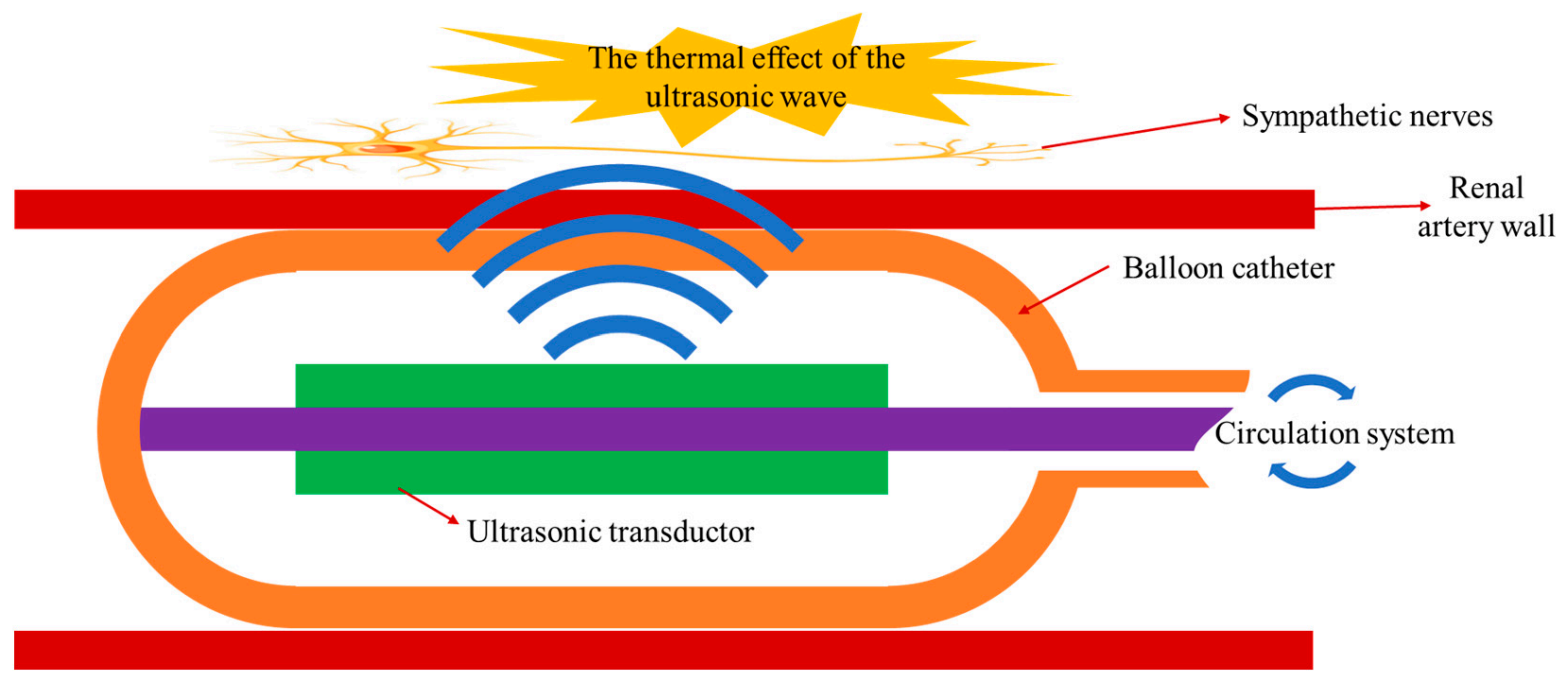
The structure and working principles of the ultrasonic RDN catheter.

Figure 1 shows a schematic diagram of the structure and working principle of the ultrasonic RDN catheter. The ultrasonic RDN catheter consists of an ultrasonic transducer, a balloon catheter, and an RDN control system. The ultrasonic transducer is mounted in the center of the balloon catheter and generates ultrasonic waves when excited by the RDN control system. Since the renal sympathetic nerves are mainly distributed around the renal artery, the ultrasonic RDN catheter is inserted into the patient’s renal artery for denervation therapy [13,16]. When the ultrasonic waves generated by the ultrasonic transducer propagate through a viscoelastic medium such as human tissue, their energy is attenuated by viscous absorption and relaxation processes in the tissue. The absorbed acoustic energy is directly converted into heat, thereby increasing the tissue temperature [29,30]. The intended mechanism is for ultrasonic waves to pass through the renal artery wall and heat the sympathetic nerves outside it sufficiently to cause protein denaturation in the nerve cells. The balloon catheter is used to isolate the ultrasonic transducer from the renal artery wall. During denervation therapy operation, the RDN control system infuses saline into the balloon catheter to fix it within the renal artery and ensures that the liquid inside the balloon is in a circulating state, with the aim of limiting the temperature rise near the renal artery wall and reducing the risk of thermal damage. When denervation therapy is finished, the control system withdraws the saline from the balloon catheter so that it can pass through the vessel. The RDN control system is used to control the operation of both the ultrasonic transducer and the balloon catheter.

### 2.1. The Design of the Piezoelectric Ultrasonic Transducer

The ultrasonic transducer is the core of the ultrasonic RDN catheter, which is used to convert electrical energy into acoustic energy. To achieve a 360° circumferential denervation region around the renal artery, a piezoelectric ceramic tube is selected as the ultrasonic transducer. Due to the better output capability, the material of the piezoelectric ceramic tube is chosen to be PZT8, a power piezoelectric ceramic material. The polarization direction of the piezoelectric ceramic tube is radial direction. When applying a voltage across the inner and outer surfaces of the piezoelectric ceramic tube, it can work in the thickness vibration mode, then generating ring-shaped ultrasonic waves.

Currently, the RDN catheters typically access the renal artery through Transradial Access (TRA) or Transfemoral Access (TFA) [31,32]. The TRA is preferred for routine diagnosis and treatment due to its minimal invasiveness. To access the radial artery via TRA, the RDN catheter typically needs to be delivered through a guiding catheter that is slightly larger but still within the tolerance of the radial artery. The size of the commonly used guiding catheter is 6F [33]. Considering that the available inner diameter of a 6F guiding catheter is approximately 1.8 mm, the outer diameter of the piezoelectric ceramic tube is selected to be 1.6 mm.

With reference to the approximately 9 MHz transducer described in patent [34], the proposed ultrasonic RDN catheter operates at a frequency of approximately 8–10 MHz. The thickness of the piezoelectric ceramic tube is determined by the frequency constant *N_t_* and the electromechanical coupling coefficient *k_t_* of the piezoelectric material. The frequency constant *N_t_* of the transducer is

(1) $$N_{t}=f_{r}t,$$

and the electromechanical coupling coefficient *k_t_* can be approximated as the effective electromechanical coupling coefficient *k_eff_*:

(2) $$k_{eff}^{2}=\frac{f_{antir}^{2}-f_{r}^{2}}{f_{antir}^{2}},$$

where *f_r_* denotes the resonant frequency of the transducer, *f_antir_* denotes the anti-resonant frequency of the transducer, and *t* denotes the thickness of the piezoelectric ceramic tube. The material characteristics of PZT8 produced by TRS Technologies are shown in Table 2 [35]. According to the frequency constant *N_t_* and the electromechanical coupling coefficient *k_t_* of PZT8 provided by TRS Technologies, the thickness of the piezoelectric ceramic tube is determined to be 0.25 mm, with the resonant frequency of 8.4 MHz and the anti-resonant frequency of 9.64 MHz. Although the output acoustic energy of piezoelectric ultrasonic transducer is the maximum at the resonant frequency, the impedance of ultrasonic transducer at the resonant frequency is also the smallest. Since the power source is used on the ultrasonic RDN catheter and connects the piezoelectric ultrasonic transducer through a long wire, the delivered electric power of piezoelectric ultrasonic transducer is limited when it operates at the resonant frequency. In addition, electrical losses can heat and damage the connecting wire. Therefore, the anti-resonant frequency of the ultrasonic transducer is selected as the operating frequency.

**Figure 2:**
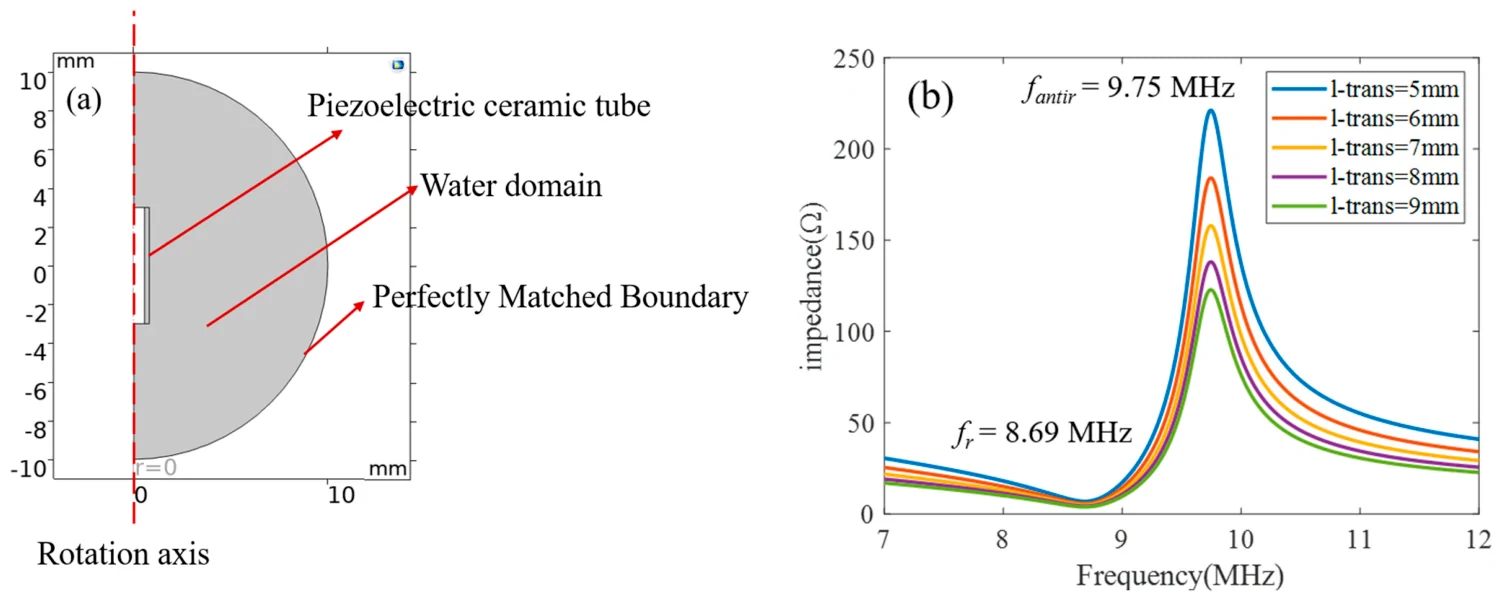

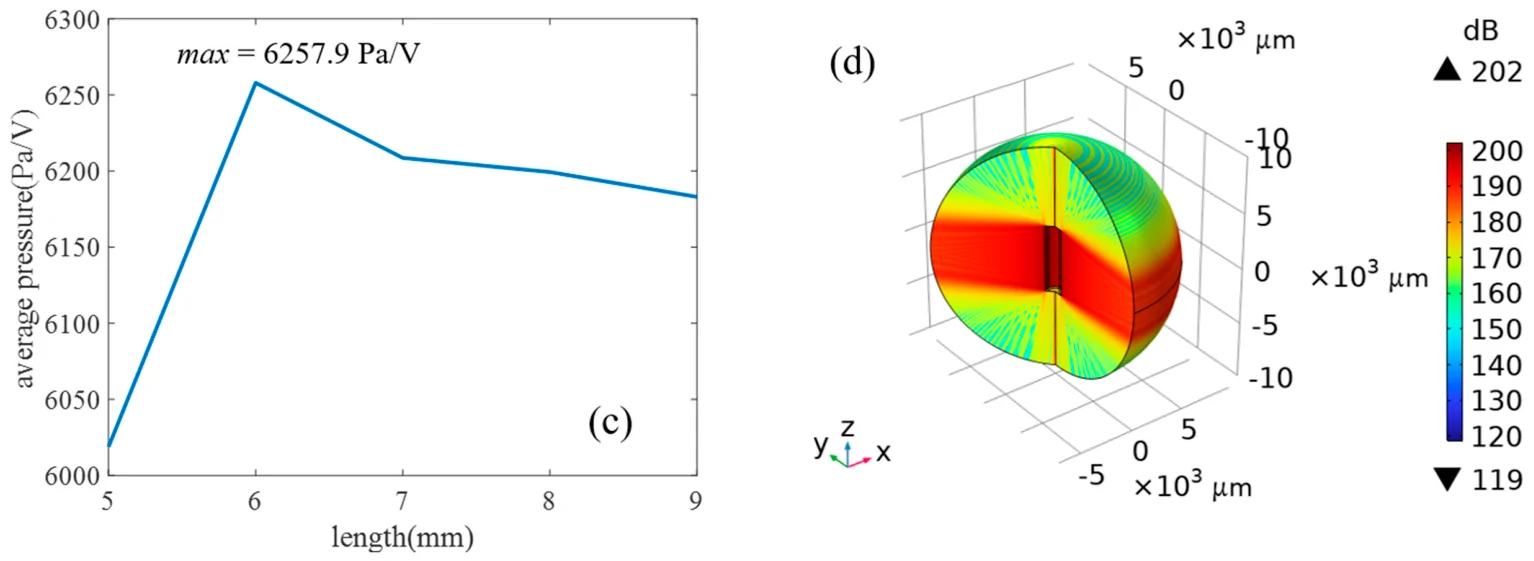
Piezoelectric ultrasonic transducer simulations. (**a**) Two-dimensional axisymmetric model and computational domains. (**b**) Impedance for tube lengths of 5–9 mm at fixed wall thickness. (**c**) Average acoustic pressure per applied voltage along a 5 mm sampling line located 4 mm from the tube; the reported maximum is 6257.9 Pa/V for a 6 mm active length. (**d**) Simulated acoustic field for the 6 mm tube at 9.75 MHz.

The ultrasonic waves output by the piezoelectric ceramic tube are also related to the length of the piezoelectric ceramic tube. After determining the thickness of the piezoelectric ceramic tube, its length is optimized using finite element simulation. The piezoelectric ceramic tube is modeled in COMSOL Multiphysics (version 6.3). To improve computational efficiency, a two-dimensional axisymmetric model is used, as shown in Figure 2a. A water domain is established around the piezoelectric ceramic tube, and a Perfectly Matched Boundary is set outside the water domain to prevent ultrasonic wave reflections at the boundary. The length of the piezoelectric ceramic tube is 5–9 mm in the simulation. Figure 2b shows the impedance of the piezoelectric ceramic tube at different lengths. It can be seen from the figure that, since the thickness of the piezoelectric ceramic tube is the same for all cases, the length of the piezoelectric ceramic tube has no effect on its resonant or anti-resonant frequencies. For piezoelectric ceramic tube lengths of 5–9 mm, the resonant frequency is 8.69 MHz, and the anti-resonant frequency is 9.75 MHz. Figure 2b also shows that as the length increases, the impedance of the piezoelectric ceramic tube decreases, which is caused by the change in the effective area of the piezoelectric ceramic tube. To evaluate the acoustic output performance of transducers with different lengths, the average acoustic pressure along a 5 mm line at a distance of 4 mm from the piezoelectric ceramic tube is calculated for each length. The simulation results are shown in Figure 2c. It can be observed that the average acoustic pressure is not simply linear with respect to length. When the length is 6 mm, the average acoustic pressure is at the maximum, which is therefore the optimal length for the piezoelectric ceramic tube. Figure 2d shows the simulation results of the acoustic pressure level of the piezoelectric ceramic tube at a length of 6 mm. It can be seen from the figure that the acoustic field of the piezoelectric ceramic tube is a uniform cylindrical shape, and the axial acoustic field exhibits weak focusing as the propagation distance increases.

### 2.2. The Design of the Balloon Catheter

**Figure 3:**
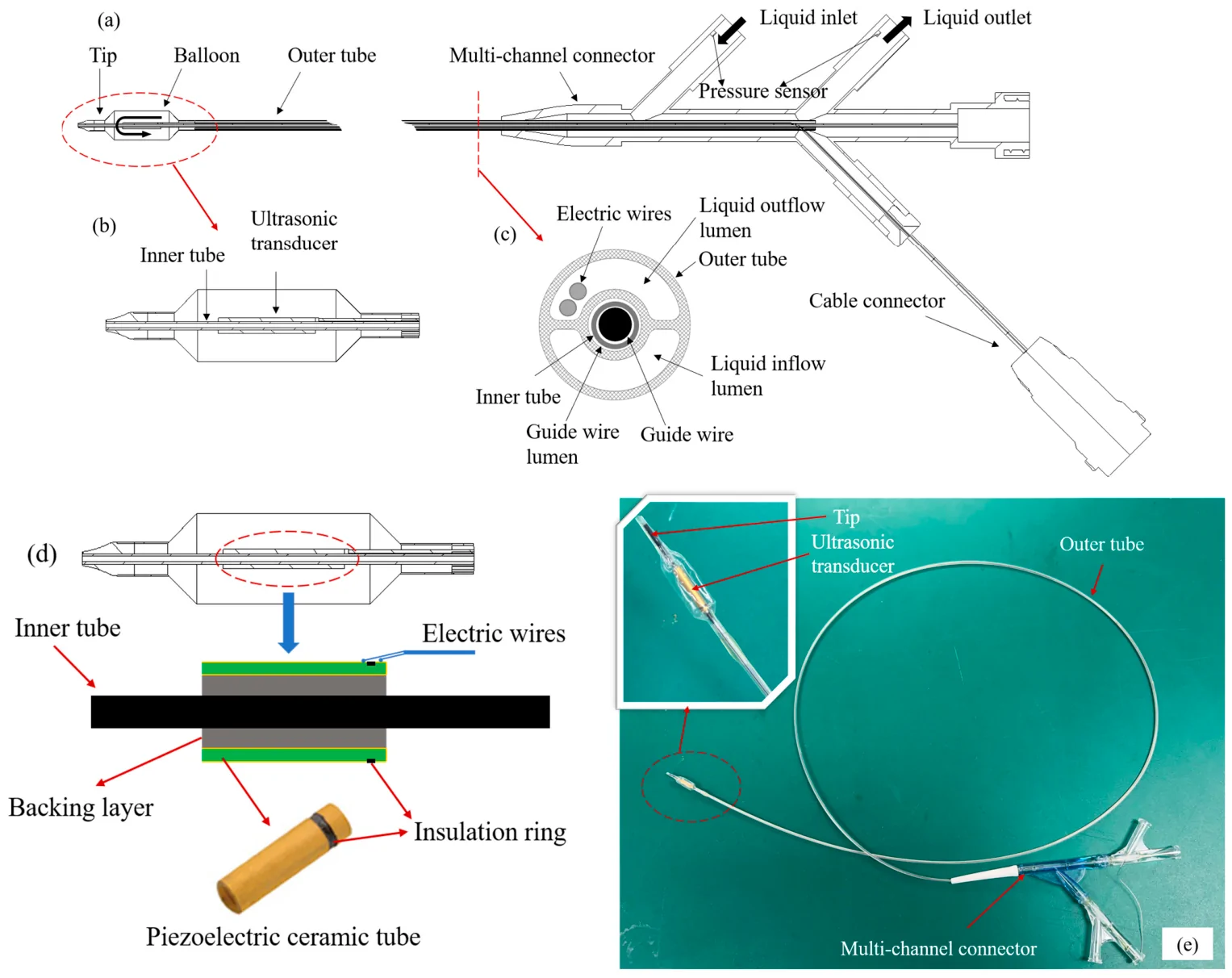
Catheter design. (**a**) The conceptual overall view of the balloon catheter. (**b**) The detail of the balloon. (**c**) The cross-sectional view of the catheter. (**d**) The assembly relationship among the piezoelectric ceramic tube, the inner tube and the electric wires. (**e**) The developed balloon catheter.

The structure of the balloon catheter is shown in Figure 3a, which consists of a tip, a balloon, a guide wire, an electric wire, an inner tube, an outer tube, a multi-channel connector, a pressure sensor, and a cable connector. The distal end of the balloon catheter refers to the end that enters the patient’s body, while the proximal end is the end operated by the physician. The balloon is located at the distal end of the balloon catheter, as shown in Figure 3b. The cross-section of the balloon catheter is shown in Figure 3c, revealing a tubular structure containing three lumens, specifically a guide wire lumen, a liquid inflow lumen, and a liquid outflow lumen. The guide wire is threaded through the guide wire lumen of the balloon catheter to guide the catheter to the target location within the body. The inner tube runs through the entire catheter to support the ultrasonic transducer while isolating the transducer from the guide wire to ensure smooth passage of the guide wire. The outer tube of the catheter is connected to the balloon and has a multi-lumen structure that allows for liquid inflow and outflow circulation. During actual use, circulating saline enters the liquid inflow lumen of the outer tube through the inlet of the multi-channel connector and is pumped through the outer tube to the balloon end. The circulating saline inflates the balloon, causing it to conform tightly to the renal artery wall. When the ultrasonic transducer operates, the heat produced by the ultrasonic transducer is absorbed through the circulating saline, then the circulating saline is pumped back through the liquid outflow lumen of the outer tube to the outlet of the multi-channel connector and subsequently to the circulating fluid reservoir. The electric wires of the ultrasonic transducer pass through the liquid outflow lumen, then connect to the cable connector. A pressure sensor is placed at each of the inlet and outlet ports of the multi-channel connector to monitor the hydraulic pressure of the balloon catheter in real time and assess the operational status of the system.

As described in Section 2.1, the dimensions of the RDN catheter are limited by the guiding catheter, and the largest part of the RDN catheter is the distal end of the balloon catheter, where the assembly relationship among the piezoelectric ceramic tube, the inner tube, and electric wires is shown in Figure 3d. The piezoelectric ceramic tube and the inner tube are isolated by a backing layer, which is a mixture of metal powder and epoxy resin. The backing layer is used to support and fix the piezoelectric ceramic tube. The piezoelectric ceramic tube has a length of 7 mm, with inner and outer diameters of 1.1 mm and 1.6 mm, respectively. The inner and outer surfaces and one end face of the piezoelectric ceramic tube are gold-plated. The inner electrode of the piezoelectric ceramic tube is led out to the outer side through the gold-plated portion on the end face, and an insulation ring on the outer side separates the inner and outer electrodes to avoid a short circuit. Meanwhile, the insulating ring divides the piezoelectric ceramic tube into an active region of 6 mm and an inactive region of 1 mm, thereby achieving the optimal length designed in Section 2.1. The electric wires lead out the inner and outer electrodes on the outer side of the piezoelectric ceramic tube. The thickness of the balloon is designed to be 0.05 mm, and the height at the connecting part between the electric wires and the electrode of the piezoelectric ceramic tube is ≤0.1 mm. As a result, the diameter of this part is designed to be ≤1.8 mm to meet the requirement of a 6F guiding catheter. Finally, the developed balloon catheter is shown in Figure 3e.

### 2.3. The Development of the RDN Control System

**Figure 4:**
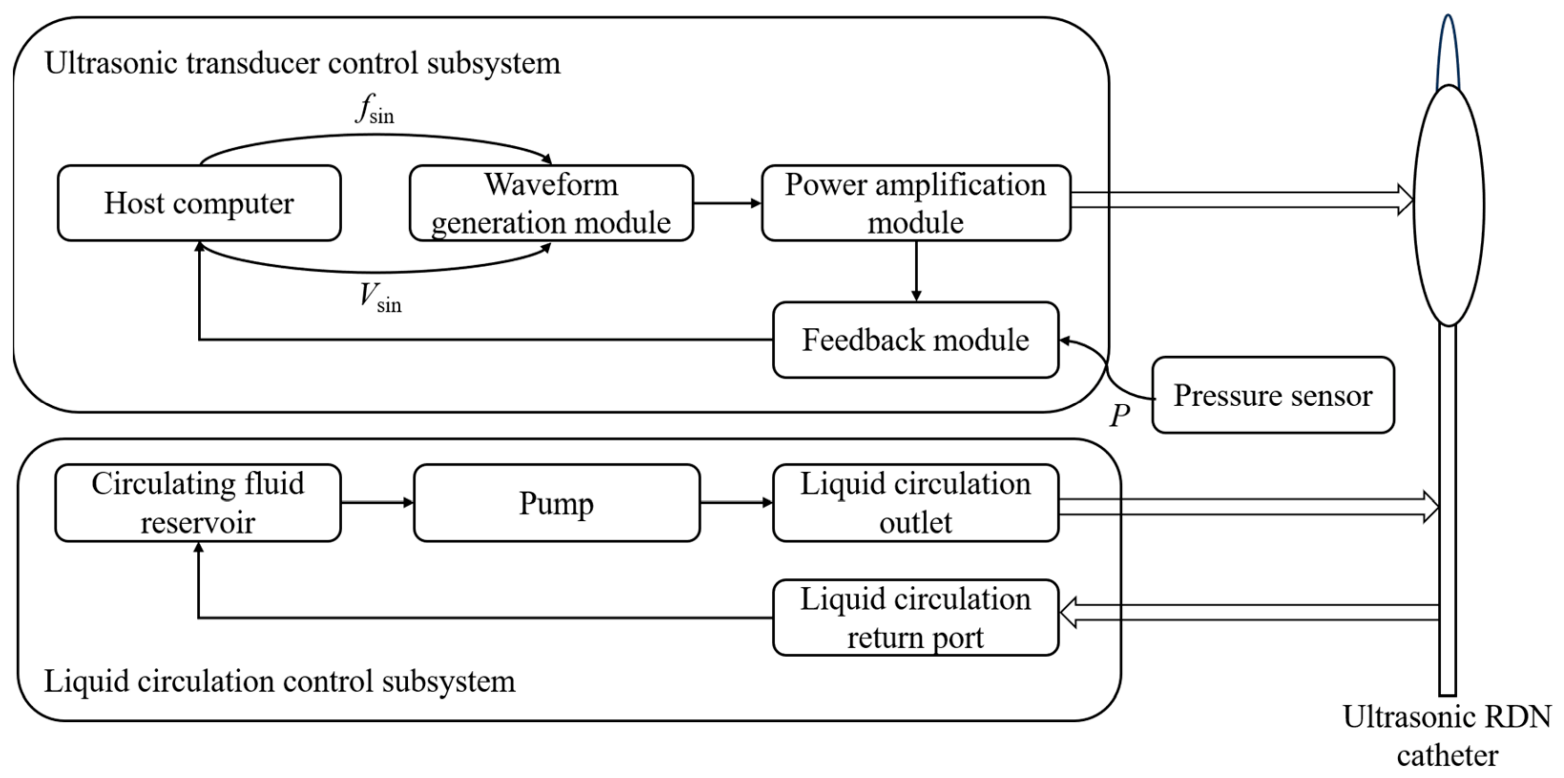
The system block diagram of the RDN control system.

The RDN control system consists of an ultrasonic transducer control subsystem and a liquid circulation control subsystem. The system block diagram is shown in Figure 4. The ultrasonic transducer control subsystem drives the ultrasonic transducer to emit ultrasonic waves for denervation therapy. The liquid circulation control subsystem controls the pump to achieve balloon deflation, inflation, and liquid circulation.

The ultrasonic transducer control subsystem comprises a host computer, a waveform generation module, a power amplification module, and a feedback module. The host computer is responsible for setting the excitation signal parameters (the frequency and voltage of the sinusoidal wave) of the ultrasonic transducer and displaying system status. It also receives data on the excitation signal voltage/current and pressure from the feedback module. The waveform generation module outputs a continuous sinusoidal wave at the required frequency and voltage as the excitation signal according to the host computer’s commands. Because the power of this signal is too low to drive the ultrasonic transducer, it is amplified by the power amplification module at a fixed gain to meet the ultrasonic transducer’s excitation requirements. The feedback module acquires the amplified excitation signal and the pressure information from the pressure sensor to the host computer for display and analysis.

The liquid circulation control subsystem consists of a circulating fluid reservoir, a pump, a liquid circulation outlet, and a liquid circulation return port. The circulating fluid reservoir stores a certain volume of saline to supply water for circulation. The pump, regulated by the host computer, provides the required water pressure. The liquid circulation outlet connects the pump to the catheter’s inlet, delivering saline to the catheter. The liquid circulation return port connects the catheter’s outlet to the circulating fluid reservoir, which is used to collect the saline exiting the catheter.

## 3. Results and Analysis

### 3.1. The Characteristic of the Ultrasonic RDN Catheter

**Figure 5:**
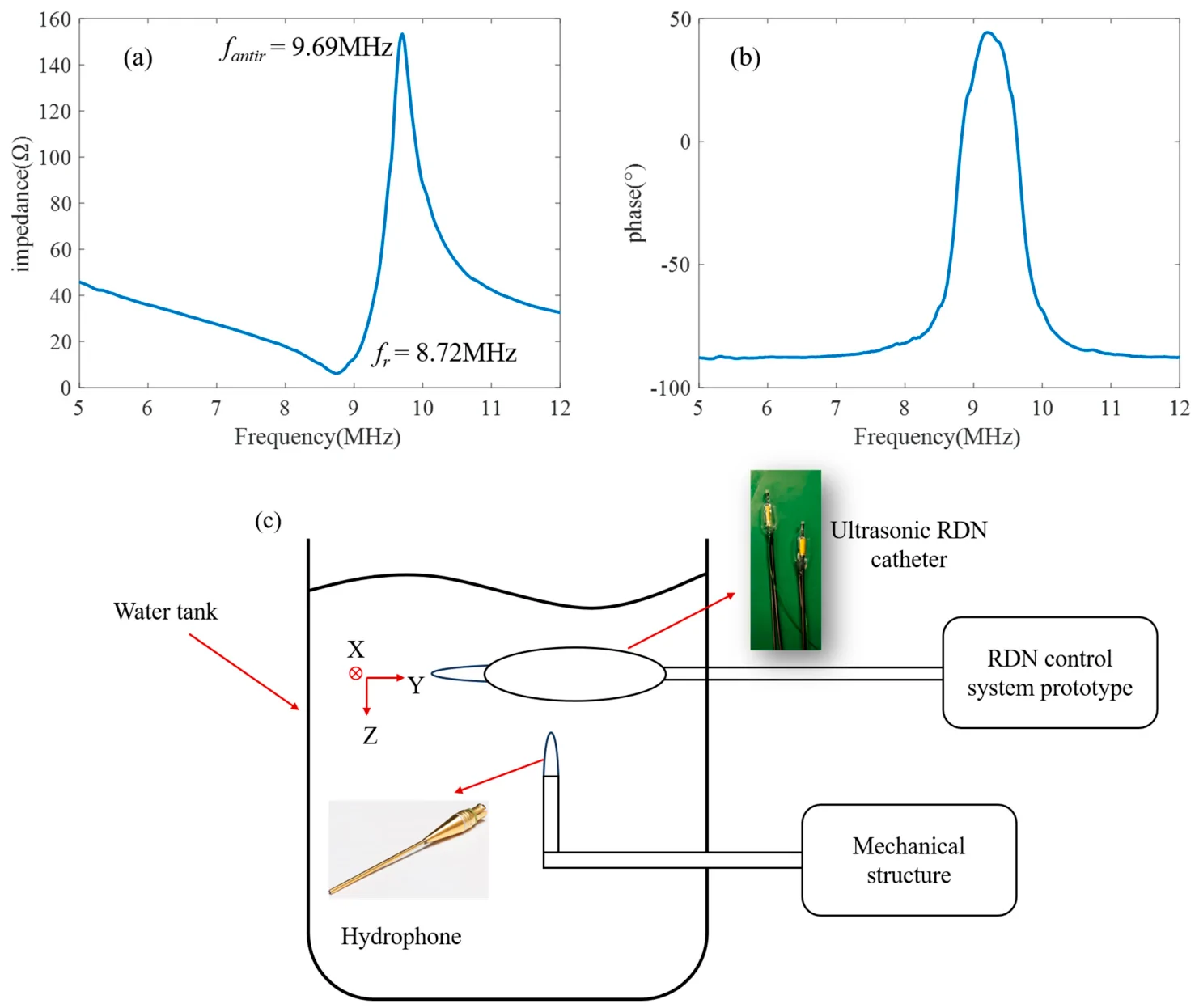

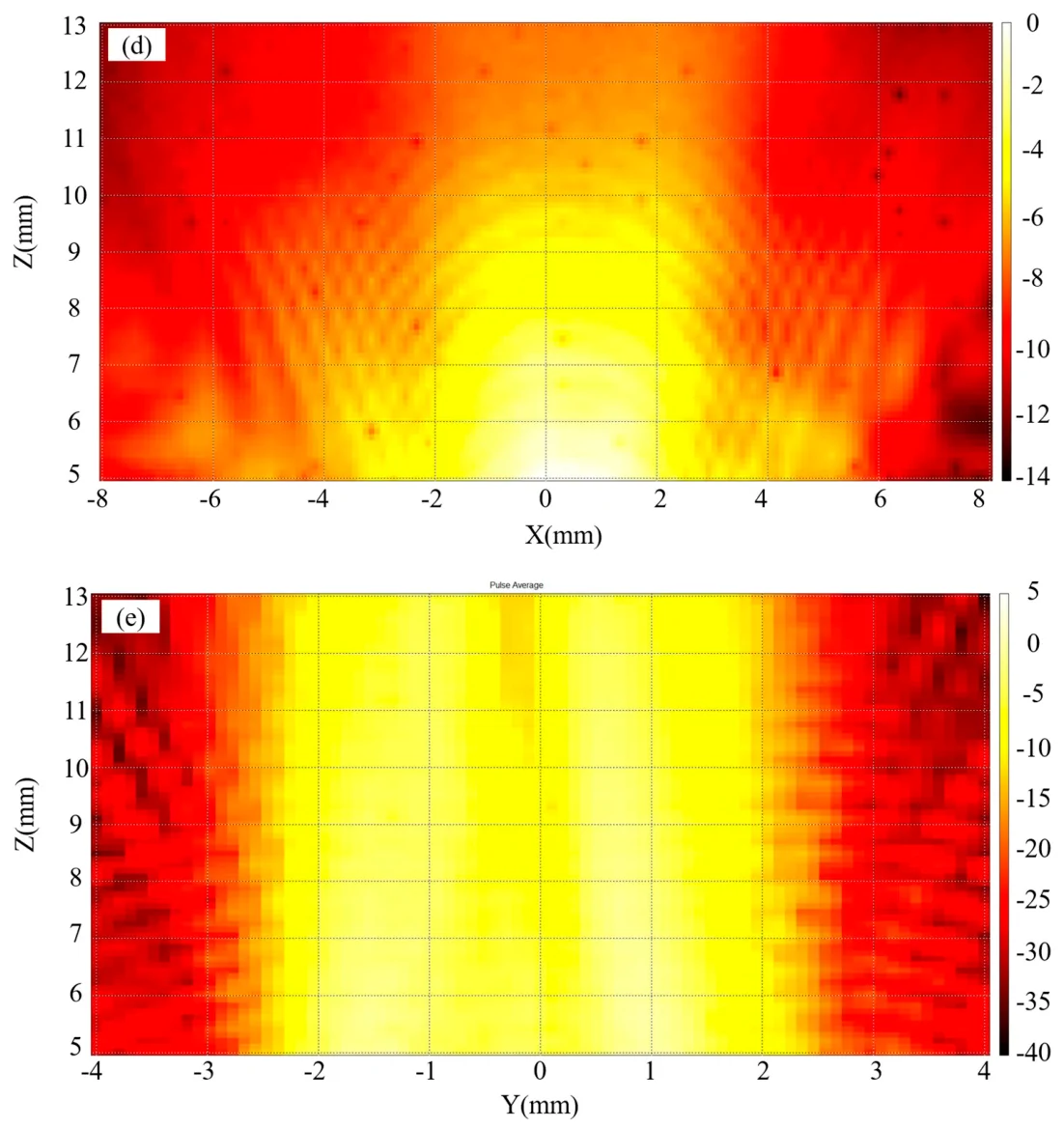
Electrical and acoustic characterization. (**a**,**b**) Measured impedance magnitude and phase; resonance and anti-resonance occur at 8.72 MHz and 9.69 MHz. (**c**) Hydrophone measurement apparatus. (**d**,**e**) Normalized acoustic pressure level results in the XZ and YZ planes.

The impedance of the piezoelectric ceramic tube used in the ultrasonic RDN catheter is characterized through an impedance analyzer (Keysight E4990A, Keysight Technologies, Santa Rosa, CA, USA), and the measurement result is shown in Figure 5a,b. As shown in Figure 5a,b, the resonant frequency (*f_r_*) of the piezoelectric ceramic tube is 8.72 MHz, the anti-resonant frequency (*f_antir_*) is 9.69 MHz, and the effective electromechanical coupling coefficient (*k_eff_*) is 43.62%. At the anti-resonant frequency, the impedance of the piezoelectric ceramic tube is 153.34 Ω, which meets the requirements of the RDN control system.

The acoustic characteristics of the ultrasonic RDN catheter are measured by a hydrophone. The measurement setup is shown in Figure 5c. The ultrasonic RDN catheter is placed at the center of a transparent water tank. During measurement, the ultrasonic RDN catheter is excited by a sinusoidal signal at the anti-resonant frequency through the RDN control system prototype with 70 W input electric power. As measured by the feedback module of the RDN control system, the effective electrical power actually applied to the transducer is approximately 50 W. The ultrasonic wave generated by the ultrasonic RDN catheter is measured through a hydrophone (NH0500). The hydrophone is driven by a mechanical structure that can scan the region of 16 mm in length (X direction), 8 mm in width (Y direction), and 13 mm in depth (Z direction). The measurement results of the normalized acoustic pressure level are shown in Figure 5d,e. As shown in Figure 5d, the acoustic field in the XZ cross-section of the RDN catheter is approximately cylindrical. The distortion of the acoustic field beyond X > 2 mm arises from the narrow acceptance angle of the needle hydrophone and the inability of the hydrophone to align with the RDN catheter during planar scanning. As shown in Figure 5e, the acoustic field in the YZ cross-section of the RDN catheter is relatively uniform, which supports circumferential acoustic delivery. The average acoustic pressure at a distance of 6–7 mm from the ultrasonic RDN catheter is measured as about 2.11 MPa.

### 3.2. Macroscopic Thermal Observations in Phantom and Ex Vivo Tissue

**Figure 6:**
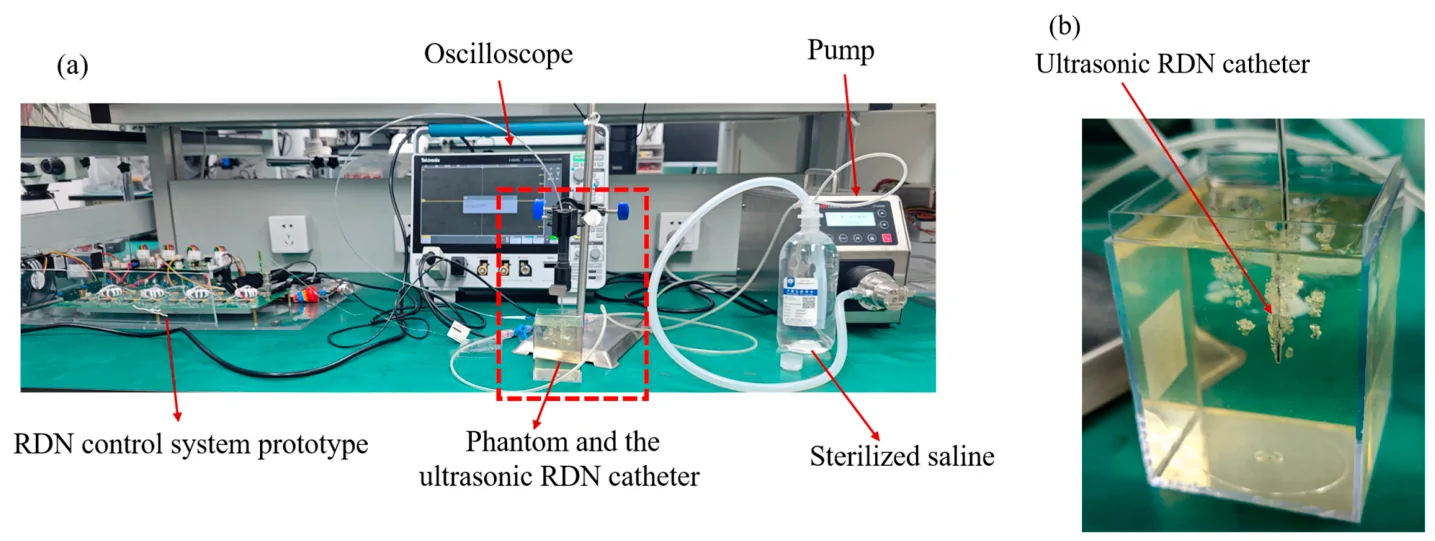

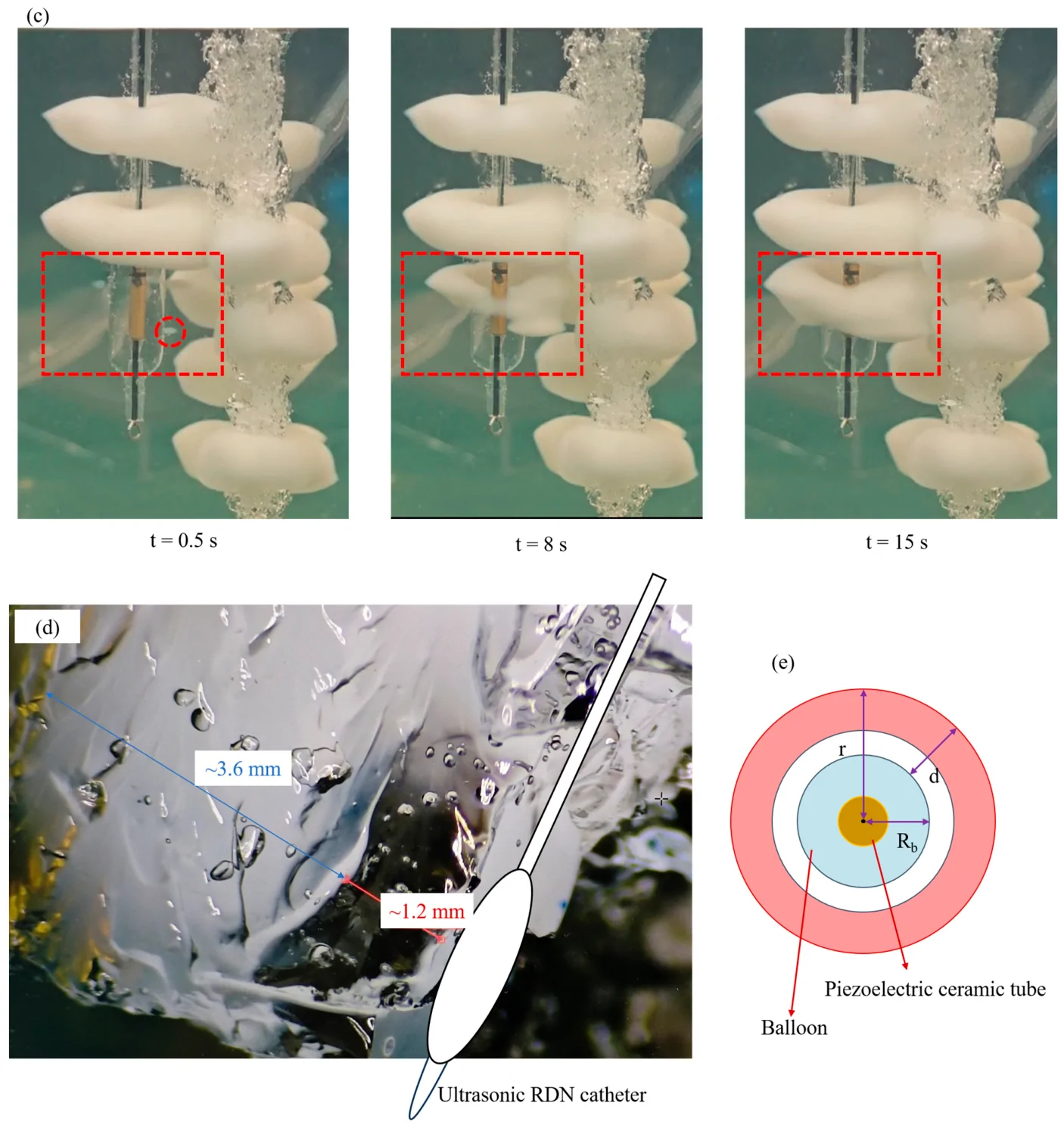
Representative phantom observations. (**a**,**b**) Apparatus and catheter placement in ONDA HIFU Phantom Gel. (**c**) Images at t = 0.5 s, 8 s, and 15 s. (**d**) Post-exposure oblique photograph retaining the original approximately 1.2 mm transparent and 3.6 mm whitened interval annotations. (**e**) Idealized transverse-coordinate schematic defining centerline radius *r*, balloon radius *R_b_*, and outward interface distance *d*.

To validate the thermal feasibility of the proposed ultrasonic RDN catheter, the experiment is conducted on a phantom. The experimental setup is shown in Figure 6a,b. The ultrasonic RDN catheter is connected to the designed RDN control system prototype, and the catheter is placed into the phantom. The phantom used in the experiments is a commercial tissue-mimicking gel (ONDA HIFU Phantom Gel) with a phase transition temperature of approximately 70 °C. This phantom is transparent at room temperature and undergoes denaturation (turning white) when the temperature reaches above 70 °C. Table 3 summarizes the experimental conditions of the phantom and ex vivo tissue experiment. Phantom Experiment 1 is used to observe the denaturation of the phantom, while Phantom Experiment 2 uses an infrared camera to record the change in phantom temperature over time.

The time for phantom denaturation is recorded during Phantom Experiment 1. The experimental results at 0.5 s, 8 s, and 15 s are shown in Figure 6c. In the phantom experiments shown in Figure 6c, three catheters are used, and three experiments are performed with each catheter. The figure shows six annular denaturation zones formed by three of the catheters. In the region marked by the red dashed box, it can be observed that the phantom begins to denature at 0.5 s after the ultrasonic RDN catheter starts working. At 8 s, a continuous 360° annular denaturation zone is formed, and when the time reaches 15 s, a denaturation region with a thickness of about 3.6 mm is achieved. Figure 6d shows the cross-sectional view of the phantom after the experiment. A measuring microscope is used to measure the distance in Figure 6d. For clarity, Figure 6d shows the approximate position of the catheter, and Figure 6e presents an idealized transverse coordinate schematic. As shown in Figure 6d, the approximately 1.2 mm transparent interval and 3.6 mm whitened interval are image-based estimates along the annotated path. If the annotated transparent interval begins at the balloon interface, this indicates that the phantom in the 1.2–4.8 mm region outside that interface has undergone denaturation and also suggests that the temperature in this region is higher than that in the region within 1.2 mm. In Figure 6e, *d* denotes the outward distance from the balloon–phantom interface, *R_b_* denotes the balloon radius, and *r* denotes the distance from the catheter centerline. For an ideal centered circular balloon with radius *R_b_* = 2.5 mm, *r* = *R_b_* + *d*. If the annotated 1.2 mm transparent interval begins at the balloon interface, the whitened interval would extend from *d* ≈ 1.2 mm to 4.8 mm; adding *R_b_* gives *r* ≈ 3.7–7.3 mm.

**Figure 7:**
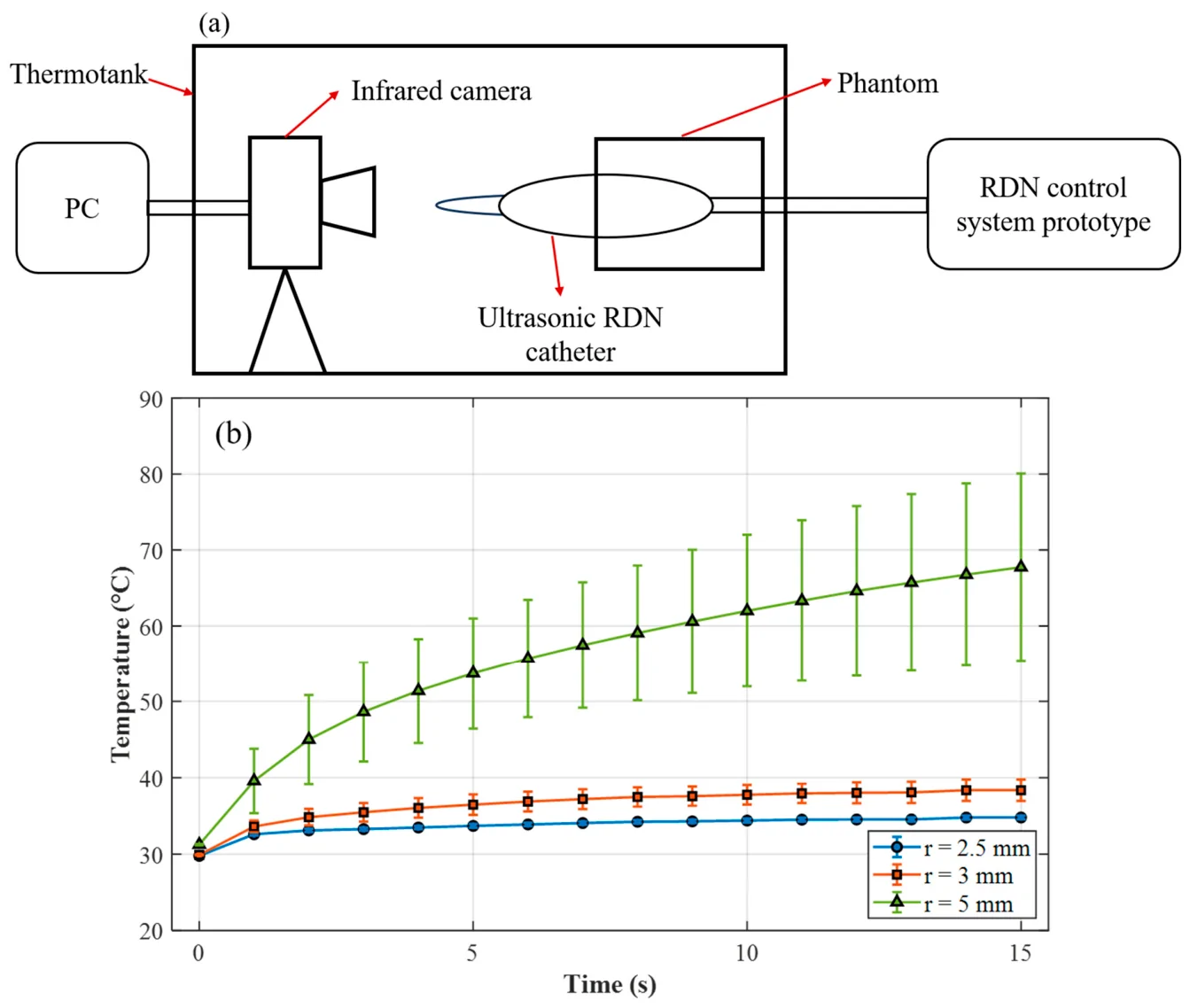
The thermal feasibility of the proposed RDN catheter in the phantom. (**a**) Temperature measurement apparatus. (**b**) Temperature–time curves near the balloon surface and within the heated region, and the error bars are calculated based on the standard deviation.

In Phantom Experiment 2, the thermal feasibility of the proposed RDN catheter on the phantom is measured via an infrared camera. The experimental setup is shown in Figure 7a. Since the infrared camera can only measure surface temperature, each catheter is placed into the phantom with its distal part extending outside the phantom so that the axial center of the balloon is located at the phantom edge, and the temperature at the phantom edge is measured. The infrared camera is aligned with the phantom edge, and the phantom is then placed in a thermostatic chamber. When the RDN control system excites the RDN catheter, the infrared camera simultaneously starts to record the temperature at the phantom edge from 0 to 15 s. The experimental conditions are shown in Table 3. Two catheters are used, and three repeated experiments are performed for each catheter. The temperatures at circumferential positions at radial distances of *r* = 2.5 mm, 3 mm and 5 mm from the balloon center are recorded. Here, *r* = 2.5 mm represents the temperature at the balloon surface, *r* = 3 mm represents the temperature in the near-balloon region and *r* = 5 mm represents the temperature in the heated region. The experimental results are shown in Figure 7b. Since the experimental setups of Phantom Experiment 1 and Phantom Experiment 2 are different, the measured temperatures are not directly interchangeable between the two setups. As can be seen from Figure 7b, the temperature at *r* = 2.5 mm is close to the medium initial temperature; the temperature at *r* = 3 mm is slightly higher than that at *r* = 2.5 mm and remains relatively low, consistent with a cooling effect of the circulating liquid; and the temperature at *r* = 5 mm is substantially higher, indicating localized phantom heating. The figure also shows that the temperature variability at *r* = 5 mm is relatively large, which may be due to catheter eccentricity, asymmetric fluid paths, and imperfect alignment between the infrared camera and the phantom.

The experimental results demonstrate that the proposed ultrasound RDN catheter is capable of forming a continuous annular denaturation region within 15 s at a centerline radius of approximately 3.7–7.3 mm (corresponding to d ≈ 1.2–4.8 mm under the interface-reference assumption above). Previous studies have shown that more than 70% of the nerves are located within 4 mm from the renal arterial lumen [2], which would correspond to *d* ≤ 4 mm in an idealized geometry with the balloon opposed to the luminal surface. Therefore, the phantom heating interval overlaps with part of this reported nerve-distribution range.

**Figure 8:**
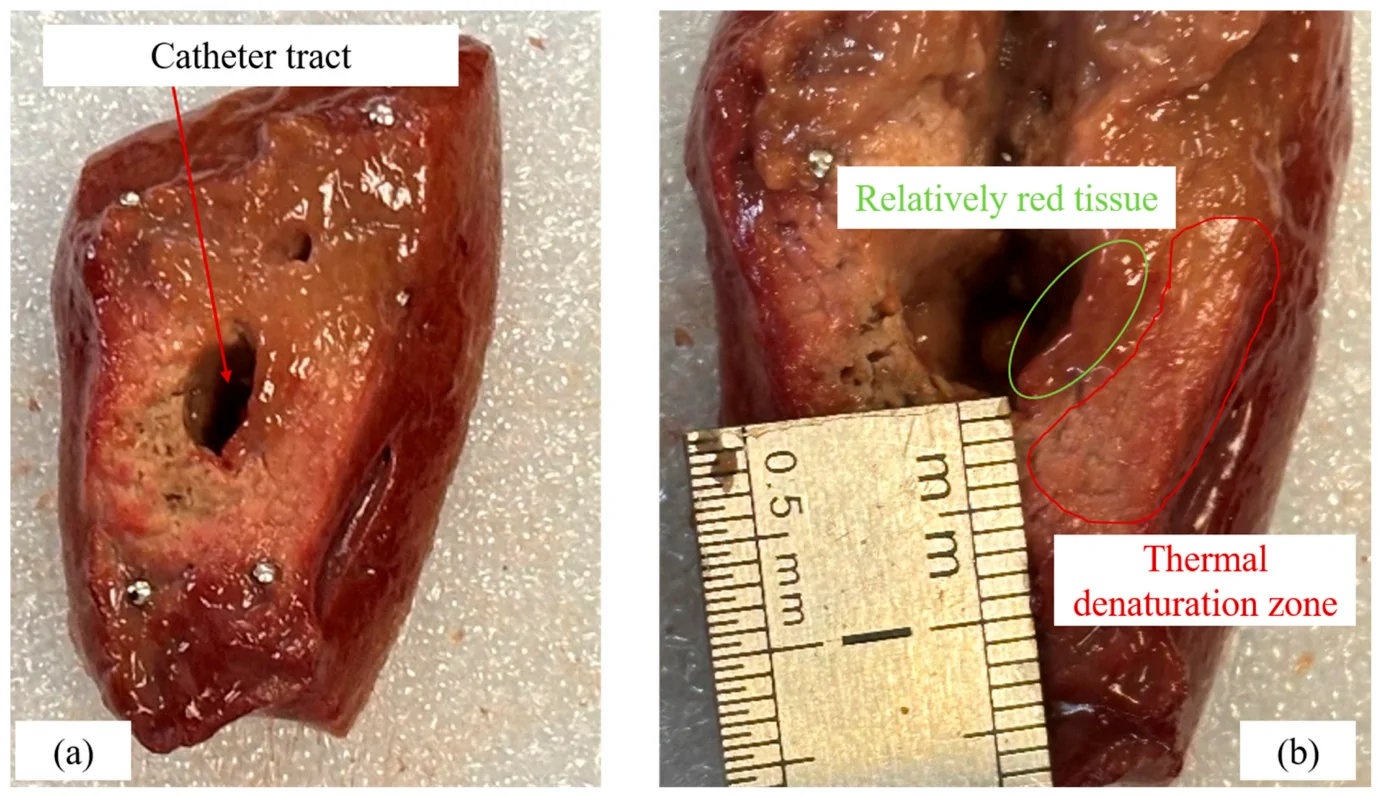
Representative ex vivo porcine liver appearance after ultrasonic wave exposure. (**a**) Section showing the catheter tract. (**b**) Enlarged view with the original ruler and contours retained; labels describe relatively red tissue and a thermal denaturation zone inferred from discoloration.

The experiments are also performed on ex vivo animal tissue. The animal tissue used is porcine liver, a commonly employed ex vivo experimental material. The porcine liver used in this manuscript is obtained from Freshippo commercial slaughterhouse in Shanghai. In this experiment, the proposed ultrasonic RDN catheter is embedded in the porcine liver. The experimental conditions are shown in Table 3: two catheters are used, and two experiments are performed with each catheter. After exposure, the tissue is sectioned for macroscopic inspection, and the results are shown in Figure 8. The displayed cut surface shows a catheter tract, adjacent relatively red tissue, and a discolored region consistent with thermal denaturation. It can be seen from Figure 8 that the reddish regions show less visible discoloration, while the white regions are consistent with thermal denaturation. The image is compatible with a section along or oblique to the catheter tract; its precise angle relative to the catheter axis is not documented, so it is not treated as a calibrated transverse section. The relatively red region is more conspicuous on one side of the displayed liver tract. Possible contributors include catheter eccentricity, local tissue contact, fluid-path asymmetry, tissue heterogeneity, and the sectioning or viewing angle.

## 4. Conclusions

This manuscript proposes an ultrasonic RDN catheter, for which the ultrasonic transducer, balloon catheter, and RDN control system are designed. The designed ultrasonic transducer is capable of achieving high-frequency radial vibration at 9.69 MHz and generating a uniform annular acoustic field, supporting circumferential acoustic exposure for the intended RDN application. The balloon catheter, under the control of the RDN control system prototype, can form a circulation system intended to limit heating near the renal artery wall. Finally, a preliminary bench evaluation is conducted on phantoms and ex vivo tissues. The experimental results show that the proposed ultrasonic RDN catheter can denature the phantom within 0.5 s and form a continuous 360° annular denaturation region within 8 s. When the time reaches 15 s, a denaturation region with a thickness of about 3.6 mm is obtained, while an adjacent transparent interval of about 1.2 mm is observed in the phantom. Ex vivo liver shows macroscopic discoloration consistent with thermal denaturation. These observations support engineering feasibility and preliminary thermal response. Next, the efficacy of the proposed ultrasonic RDN catheter will be further investigated in ex vivo tissues with nerves, followed by in vivo animal experiments and long-term stability studies, to further validate its potential for clinical application.

## Figures and Tables

**Table 1:** Comparison with prior art.

Product	Symplicity Spyral	Netrod	ReCor Medical Paradise	This Work
Denervation method	radiofrequency	radiofrequency	ultrasonic	ultrasonic
Access route	TFA (Transfemoral Access)	TFA	TFA *	TRA (design target)
Exposure pattern per activation	4 points	6 points	360° circumferential region	360° annular phantom response
Active fluid cooling	No	No	Yes (liquid circulation)	Yes (liquid circulation)
Exposure duration	≥60 s	≥60 s	7 s	15 s (phantom)

* According to the FDA approval announcement, the ReCor Medical Paradise system reaches the renal artery via TFA [28]. Radial-access RDN has also been reported with the ReCor Radiance catheter [26], which is a different device. In this manuscript, the FDA approval announcement is used as the reference.

**Table 2:** The material characteristics of PZT8.

Characteristics	Quality Factor (*Q*)	*N_t_*	*k_t_*
Value	900	2100 Hz·m	0.49

**Table 3:** Experimental conditions of phantom and ex vivo tissue experiment.

Experiment	Phantom Experiment 1	Phantom Experiment 2	Ex Vivo Liver Experiment
Medium	ONDA HIFU Phantom Gel		Commercially sourced porcine liver
Excitation	9.69 MHz sinusoid; 70 W input electrical power; approximately 50 W effective electrical power at the transducer		
Treatment duration	15 s		
Medium initial temperature	25 °C	30 °C	25 °C
Saline initial temperature	25 °C	30 °C	25 °C
Liquid circulation parameter	flow rate 80 mL/min, input pressure 4.25 atm, output pressure 0.25 atm, calculative input flow velocity 2.67 m/s		
Independent units	3	2	2
Replicates per unit	3	3	2

## Data Availability

The original contributions presented in this study are included in the article. Further inquiries can be directed to the corresponding authors.
